# Medical and Dental Examination of Children in the Public Schools

**Published:** 1913-09

**Authors:** J. L. Bowman

**Affiliations:** Union Springs, Ala.


### MEDICAL AND DENTAL EXAMINATION OF CHILDREN IN THE PUBLIC SCHOOLS.

By J. L. Bowman, M. D., Union Springs, Ala.

This paper does not purport to be a review of literature on this subject nor can we discuss the advantages and results as shown by the reports from many cities and towns throughout the country which have adopted such measures, but rather to give the methods we have used for two years and the impressions this work has made and more particularly to answer the many inquiries we have received regarding our work, the substance of which is "how did you establish a satisfactory system of medical and dental examination of the children in your schools."

In any public health propaganda a systematic effort to educate the public must be made. Only in this way can we secure the popular approval and support so necessary for efficient work. Show the people the needs and tell them what can be accomplished and they will support the work. If physicians will but take the time to point out the minor contagious diseases for which a physician is not consulted to the patrons of the school, existing as sources of infection to all other school children. Say Itch, probably the least harmful though ever-present contagion to which school children are subjected, and the people are ready to take any reasonable steps for its prevention The more important work, however, of the inspector is in dealing with the milder forms of such diseases as diphtheria, scarlet fever, mumps, etc., where no physician has been called and to point out to the parent such conditions as adenoids, hook worms grandulated eyelids and other conditions which are hindering the child's normal development. If the profession will make an effort to show these needs to the people they will not only be satisfied with the institution, but will demand that it be instituted and furthermore be willing to pay the price.

Insist on your dentist telling the people something about the condition of the mouths of school children and what effect such conditions will have on the child's development. We all know that bad teeth prevent mastication. We know that pus from abscessed teeth swallowed with food must injure the child's health more or less. We know too that a mouth full of good teeth which 'don't hit' will never be of much service to the owner and consequently the development, mental and physical, will be deficient. It is equally true that not one parent in ten pays any attention to the teeth of a child of school age unless they ache, usually too late for their preservation.

Enlist the aid of the newspapers. The most of them are willing to aid in waging a campaign for the public good.

Interest the city council or commission and of still more importance, interest the school board. If either of these bodies have members too old to be interested in the welfare of the rising generation of men and women, in the words of the poet, 'Take him out without making a noise' and I may add, give his place to one of the boys.

When the public and the powers that be have ordered the work done the man to do it is possibly a more momentous question. It can not be done for the money that is in it and after the contract-practice fashion, for in that case the inspector would only do what he is paid for which will not be very much. The man who does this work must be sufficiently interested to look after and guard those children as he would his own family and it may be well enough for him to have a part of his family among them. He must recognize the fact that his duties are educational as well as an officer of the public health. As an educator, think what the effect on the citizenship of Alabama would be in a few years if school inspectors were to carry the campaign of education now being waged by the state's efficient health officer into the schools of Alabama so that each pupil would be as familiar with the public health rules from the registration of vital and mortuary statistics, to the polution of soil and sources of water supply as they are with the spelling book.

Pay the school inspector for his services if it can be done, otherwise he will do it for humanity which every true physician wishes to benefit. The careful, conscientious, faithful school inspector will secure the approval of the public regardless of the opposition that may exist at the beginning who will sooner or later be glad to pay for his services.

Dr. Osler says that more people are dying from the effects of bad teeth than the effects of alcohol. It therefore behooves the medical profession to call upon our dental brethren, to come forward and help up to look after the interest of public health. I do not believe the dental profession as a whole is wide awake to the demands and responsibilities which this progressive age places upon them and when they fail to fall in line we must call upon them.

Our system of school inspection has been organized and at work now for two years and I dare say that there is scarcely a citizen of the town who will not point with pride to the results.

A few days prior to the opening of school we begin the examination of the pupils for entrance. We inspect carefully the eyes, throat and integument of face, hands and fore-arms for evidence of infectious and contagious diseases, adenoids and other conditions that may demand a more detailed examination. Along with name, age, etc., we carefully note the physical condition on this entrance certificate which is made in duplicate.

Name ....................................

Residence ....................................

Parent or Guardian ............................

Grade ............ Sex............ Age........

Physical Condition.............................

.........................................

may be allowed to enter school.

....................M. D.

Date .............191.. Physician School Inspector

One copy is given to the pupil who presents it to the dental inspector. The dental examination is then made and this certificate showing the condition of the child's mouth is given to the pupil.

Report of UNION SPRINGS PUBLIC SCHOOL on
Oral Hygiene and Prophylaxis

Grade............. Date.................

Name of Pupil ....................................

Age ............ Sex.............. Race..............

No. of Teeth that need filling ............

No. of Teeth filled ............

Teeth need straightening, Yes. No.

Gums need treatment, Yes. No.

Has child any abscesses in mouth, Yes. No.

Root or decayed Teeth need extracting, Yes. No.

V-shaped arch, Yes. No.

| | | |
|---|---|---|
| Does child breathe through mouth or nose? | .......... | |
| Has child tooth brush? | Yes. | No. |
| If yes, is it daily used? | Yes. | No. |
| Teeth need cleaning by a dentist? | Yes. | No. |
| Does child need any other treatment? | Yes. | No. |

To parent or guardian:—

This is a true examination of your child's mouth and we trust, without delay, you will immediately have this trouble properly attended. If you wish your child to have good health, pretty teeth, and be bright in its studies and save hours of suffering, be certain that the child uses the tooth brush after meals. Remember clean teeth do not decay.

.................................... Examiner.

Both medical and dental certificates are taken home to the parents who are urged to examine them. Both must be presented to the principals of the schools before the pupil is permitted to enter. From the duplicate copy of the dental certificate the dental examiner enters in a permanent record in detail the condition of each child's mouth.

The medical inspector from the duplicate certificate retained and reports from the dental examiner and superintendants makes up his permanent record showing name, age, physical condition, parent or guardian, residence, absence, condition of mouth, grade of work.

In Alabama the state board of health has on record the data regarding a man's birth and death, but think what the value of such a record of the school life of every child in the schools of the state would be, to the medical profession, to the dental profession, to educators and to sociologists. In a few years our statistics would show with mathematical precision the effect of physical defects on the mental powers of the child. We would have a physical and mental census so to speak each year.

Once each month or more if conditions demand, the medical inspector visits the schools and examines the pupils in somewhat similar manner as at the time of entrance. When a case of contagious disease is found the following form is used, one copy for examiner's record, one for the teacher and one to be sent home by the pupil to the parent or guardian.

MEDICAL INSPECTION OF SCHOOLS
Union Springs, Alabama
..................191..
School ......................Grade......
Name of Pupil ...................Age....
Home Address .........................
The above named Pupil is hereby ordered to discontinue attendance at School temporarily for the following reasons:
.......................................
.......................................
......................M. D.
Hand to Pupil Excluded. Physician School Inspector

Should anything appear which prevents the child from doing good work, this blank is used and the examiner's record retained on a stub.

UNION SPRINGS PUBLIC SCHOOLS
..................191..

To Parent or Guardian:
...........................is suffering from
.......................................
.......................................
Which prevents from doing school work as well as would do if relieved and retarts development into a healthy You will do well to consult your family physician regarding condition. This does not debar from school.
....................M. D.
Physician School Inspector.

The inspector advises the pupils in matters of personal health and hygiene and looks after the sanitary condition of the school house and premises. Should any pupil be absent for three consecutive days he or she is not permitted to return to school until the school inspector has made an examination and given a certificate of admission.

Union Springs, Ala.,..................191..

.......................................

may be allowed to return to school

..................M. D.

Physician School Inspector.

On the stub of this permit the inspector notes name, date and cause of absence.

The school board of Union Springs requires a normal fee for both the entrance examination and certificates for readmission and finds that it serves well the primary object, namely, effecting a more prompt entrance and diminishing the number of absentees.

Now as to the results of two years of this work, I have not the time to go into details, but if you had a list of the adenoid operations performed on school children from Union Springs you would see we were doing something. We have reduced scabes from an epidemic of rather pandemic to a rarity. We have, during the past term which opened at a time when there was a wide-spread epidemic of diphtheria, generally in such mild form that no physician was called, by persistent examination of every suspicious throat and proper quarentine, so controlled the disease that no new cases developed in the schools after the first month and no new cases in the town after the second. An epidemic of mumps was held to half a dozen cases in the white school though among the negroes the disease made more headway. Right here let me say that in the matter of medical and dental inspection, look well to the negro schools as they are a source of infection of many epidemics of contagious diseases and I believe one factor that prevented our controlling the epidemic of mumps was the close proxmity of a private negro school over which we had no supervision. I mention these particular things because they do not come within the scope of the work expected of the health officer or the measures adopted are not at his command. Scarlet fever, small pox, and the severer cases of diphtheria were his charges.

Now last but not least, I predict that within a half decade you are going to hear that the children of Union Springs have better teeth, cleaner mouths and therefore better digestion and will make physically better men and women than those of any other town in the state except the one which does the work which is now being done here.

One of the speakers stated that he had read a paper of like nature before the 4th Congressional District of Georgia Medical Society and that he found it necessary to request statistics from superintendents of public schools and commissioners of the county seats composing the fourth congressional district. He found that in the fourth congressional district there was not a school that had a systematic inspection of the schools, mental or dental; some of the reports stating that such inspections as had been made were by the chief of police, of some officer of the city council, or something like that; and stated that he wanted to agree with Dr. Bowman in his reference to the negro school children and the lack of interest and sanitation around negro settlements; that there was no sanitary inspection or precautions where they were most needed, that is, around negro settlements, and that in epidemic or contagious diseases, these settlements contributed 75 per cent. of the cases.

